# The Hippo Pathway: A Master Regulatory Network Important in Cancer

**DOI:** 10.3390/cells10061416

**Published:** 2021-06-07

**Authors:** Qiuping Liu, Xiaomeng Liu, Guanbin Song

**Affiliations:** 1 Institute of Neuroscience and Translational Medicine, College of Life Science and Agronomy, Zhoukou Normal University, Zhoukou 466001, China; liuqp@cqu.edu.cn (Q.L.); lxmxm_99@126.com (X.L.); 2 Key Laboratory of Biorheological Science and Technology, Ministry of Education, College of Bioengineering, Chongqing University, Chongqing 400030, China

**Keywords:** Hippo pathway, signal transduction, metabolic regulation, cancer therapy

## Abstract

The Hippo pathway is pervasively activated and has been well recognized to play critical roles in human cancer. The deregulation of Hippo signaling involved in cancer development, progression, and resistance to cancer treatment have been confirmed in several human cancers. Its biological significance and deregulation in cancer have drawn increasing interest in the past few years. A fundamental understanding of the complexity of the Hippo pathway in cancer is crucial for improving future clinical interventions and therapy for cancers. In this review, we try to clarify the complex regulation and function of the Hippo signaling network in cancer development, including its role in signal transduction, metabolic regulation, and tumor development, as well as tumor therapies targeting the Hippo pathway.

## 1. Introduction

During tumor development, cancer cells are exposed to dynamic changes in the tumor microenvironment and availability of nutrients [1,2,3]. Hence, cancer cells need to adapt their physiological processes and metabolism to these changes so as to maintain, survive, proliferate, and even undergo behavioral changes such as invasion and metastasis [4,5]. This is a complex signaling cascade, including cellular perception and transduction of stimuli in the microenvironment, as well as intracellular metabolic reprogramming to support the high demand for energy and building blocks [6]. The involved signaling cascades are affected by some transcription factors and associated pathways. One such pathway is the Hippo signaling pathway, which regulates gene expression in response to changes in extracellular and intracellular cues, leading to changes in cell behavior [7].

The Hippo pathway is a unique signaling module that regulates cell-specific transcriptional responses and responds to a wide range of intrinsic and extrinsic cues [8,9,10]. Based on the ability of the yes-associated protein/transcriptional coactivator with a PDZ-binding motif (YAP/TAZ) to regulate signal transduction, metabolism adaptation, and phenotypic changes, it is expected that the Hippo signaling pathway will function as a central hub for cancer development. In this review, we will introduce the regulatory role of the Hippo pathway in tumor development, signal transduction, and metabolism, and discuss possible cancer treatment strategies targeting the Hippo pathway.

## 2. Role of Hippo Signaling in the Development of Cancer

Hippo signaling, first discovered in Drosophila, has been implicated as a key regulator of organ size based on its important roles in regulating cell proliferation and apoptosis [11,12,13], and in regulating tissue-specific stem cells. The Drosophila Homolog of YAP, Yorkie (Yki), was found to act as a critical target of the Wts/Lats protein kinase as well as a potential oncogene, and the Hippo signaling pathway coordinately regulates cell proliferation and apoptosis by inactivating Yki [14]. Evidence also demonstrates the critical role of the Hippo pathway in cancer stem cell biology, including EMT, drug resistance, self-renewal, and differentiation [15,16]. The role of the Hippo pathway in inhibiting cell growth, proliferation, promoting apoptosis, and regulating stem cell biology is the key to tumor inhibition [17].

Excessive activation or deletion of the Hippo signaling pathway will lead to abnormal cell growth and dysregulation of tissue and organ homeostasis, which will further lead to abnormal tissue and organ development, and impaired regeneration or tumorigenesis [18,19,20,21]. In fact, studies have demonstrated the important role of the Hippo pathway in the development of many kinds of cancer [22,23]. Numerous correlations between aberrant Hippo pathway protein expression and the cancer’s clinical stage (evaluated comprehensively according to tumor size, lymph node status, and distant metastasis) have been reported (as reviewed in [24]). Consistently, studies have reported that inhibition of the Hippo pathway promoted cell proliferation, migration, invasion, and the development of hepatocellular carcinoma [25,26]. As reviewed recently, the high expression of YAP/TAZ could promote breast cancer metastasis, and targeted therapy against YAP/TAZ can effectively block breast cancer metastasis [27]. These results establish a clear role for the Hippo signaling pathway in cancer progression.

As profiled by The Cancer Genome Atlas (TCGA), Hippo pathway genes such as large tumor suppressor1/2 (LATS1/2) and YAP were somatically mutated in 10% of 9125 tumors across 33 cancers [28]. The defect of mammalian sterile 20 like1/2 (MST1/2), an important component of the Hippo pathway, would lead to YAP activation, sustained liver overgrowth, and the eventual development of hepatocellular carcinoma and cholangiocarcinoma [29]. Studies have focused on how upstream inputs affect the activity of the Hippo signaling pathway, how it functions, and the contribution of its dysregulation to cancer development. Research on the mechanism of the Hippo regulatory network in cancer development has made some achievements, and its role in signal transduction and metabolism regulation is the most understood at present.

## 3. Role of Hippo Signaling in Mechanotransduction

During tumorigenesis or metastasis, cells are in a complex microenvironment and constantly respond to biochemical cues and mechanical stress from the microenvironment. These biochemical and mechanical signals are converted into intracellular signals through signal transduction to regulate the biological behavior of cells [30]. In recent years, studies have shown that during tumor development and growth, Hippo signaling plays an important role in signal transduction, particularly in mechanotransduction [31]. The key components of the Hippo cascade, including MST1/2, LATS1/2, and YAP/TAZ, constitute a phosphokinase axis, which regulate the downstream effectors to maintain homeostasis and prevent tumor growth [32].

**Figure 1:**
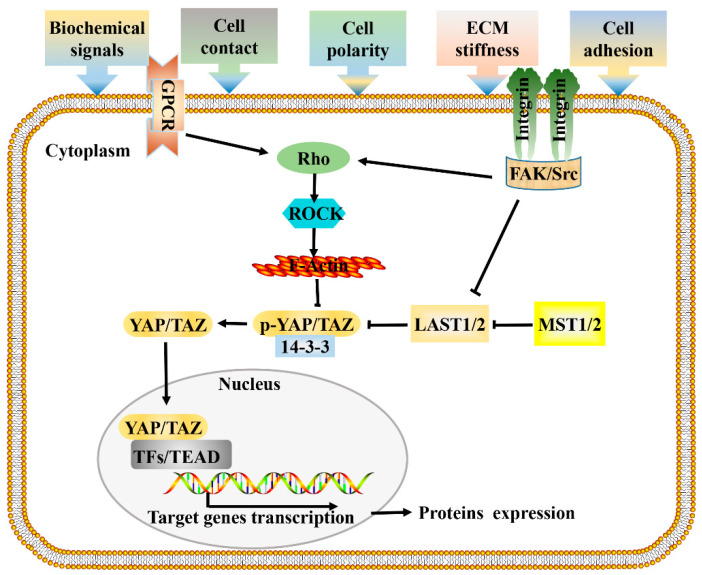
Signal transduction network of Hippo pathway. Cells respond to mechanical forces, cell polarity, and adhesion signals by adjusting their tensional state and actin dynamics to regulate the activity of Hippo pathway components. When a cell adheres to a larger area or grows on a harder ECM, causes the activation of integrin signaling, and promotes the assembly of FAK/Src complex, which inactivate LATS1/2 and facilitate the polymerization of F-actin cytoskeleton via Rho-GTPases, F-actin then induces the dephosphorylation and guides the nuclear translocation of YAP/TAZ. GPCR signaling responds to a variety of activators (energy, proteins, lipids, sugars), and performs the same function by acting on RHO-GTPases. Hypo-phosphorylated YAP and TAZ accumulate in the nucleus where they can bind to various TFs, most notably the TEAD family, to direct gene expression changes that control a range of biological events. Pointed and blunt arrowheads indicate activating and inhibitory interactions, respectively. FAK, Focal adhesion kinase; GPCRs, G-protein-coupled receptors; LATS, Large tumor suppressor; MST, Mammalian sterile 20 like; Src, steroid receptor coactivator; TEAD, TEA domain protein; TFs, transcription factors; YAP/TAZ, yes-associated protein/transcriptional coactivator with PDZ-binding motif.

As reported by Sansores-Garcia, the Hippo pathway was first linked to the cytoskeleton, as the activity of Yap is modulated by changes in F-actin [33]. Since then, cytoskeletal rearrangement and intracellular signal transduction have attracted extensive attention in the study of the mechanisms of cell regulation by mechanical factors [34]. According to previous research, mechanical forces from the microenvironment are transmitted through membrane receptors, actin cytoskeleton, and the nuclear membrane, and then affect gene transcription in the nucleus [35,36], ultimately determining cell fate and influencing tumor progression. Mechanical stimuli such as extracellular matrix (ECM) stiffness, cell morphology, and cell density would cause changes in cell geometry and cytoskeleton tension [37,38]. Studies have shown that changes in the activity or state of the cytoskeleton are involved in the regulation of the Hippo signaling pathway; knocking down or interfering with the distribution of the cytoskeleton leads to Hippo changes, thus revealing the relationship between Hippo signaling and the cytoskeleton [39,40,41,42]. At present, it is still controversial whether mechanical factors regulate YAP/TAZ through the non-classical Hippo pathway (actin cytoskeleton -YAP) or the classical Hippo pathway (MST- Lats -YAP) [43,44]. Earlier studies have found that the mechanotransduction mediated by YAP/TAZ required Rho GTPase activity and tension of the actomyosin cytoskeleton but was independent of the Hippo-LATS cascade [38]. In addition, it was reported that the depletion of LATS1/2 did not rescue YAP/TAZ inhibition through a physically soft environment [33,45]. These studies demonstrated that mechanical cues can affect YAP/TAZ activity independent of LATS (non-classical Hippo pathway). Inconsistently, the LATS1/2-dependent regulation of YAP/TAZ activity by stress fiber (F-actin) formation has been reported [46], demonstrating that mechanical cues can also affect YAP/TAZ activity in a LATS-dependent way (classical Hippo pathway). In addition, when cells are exposed to energy stress or certain biochemical stimuli, the signal transduction pattern is similar. These research results unraveled how external environmental signals control related gene expression through classical and actin cytoskeleton-regulated Hippo signaling, helping to more clearly depict the mechanism of Hippo pathway mediated signal transduction (Figure 1).

## 4. Hippo Signaling in Cancer Metabolic Reprogramming

Metabolism is a fundamental function of cells that can be reprogrammed to meet the energy and material needs of cells through the regulation of signaling pathways. In turn, metabolic pathways or metabolites can modulate a network of signaling pathways, allowing cells to coordinate their metabolism and behavior in an integrated manner [47]. In the past several years, increasing studies have provided appreciation and understanding of how Hippo signaling controls cellular and organismal metabolism, and the diverse mechanisms through which metabolites and metabolic signals, in turn, influence Hippo signaling [8,47]. The Hippo signaling pathway is a highly conserved tumor suppressor pathway, which was identified as emerging nodes in the coordination of nutrient availability with cancer development and tissue homeostasis (Table 1).

### 4.1. Regulation of Metabolism by Hippo Signaling

**Figure 2:**
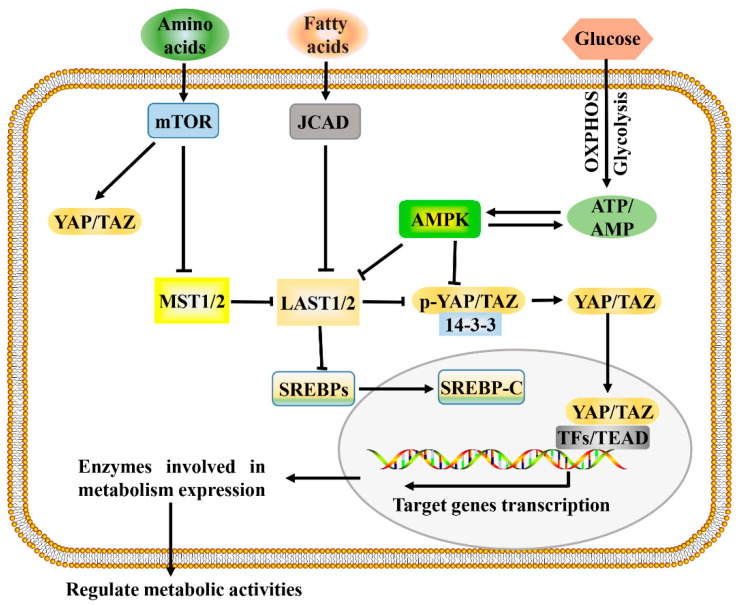
A proposed model for Hippo pathway regulation by metabolism and Hippo pathway targets for metabolism. Metabolism can regulate or be regulated by the Hippo pathway through different mechanisms. In a nutrient-rich environment, glucose through glycolysis and mitochondrial OXPHOS inactivates AMPK by increasing the ATP:AMP ratio; in an energy stress environment, active AMPK inhibits YAP/TAZ by direct phosphorylation and/or activating LATS1/2. Amino acids induce the activation of mTOR, which also activates YAP/TAZ through various mechanisms. Fatty acids increase the expression of JCAD, which inhibits LATS2. Hippo core kinases MST and LATS regulate lipogenesis, as LATS2 can directly bind to SREBP precursors (SREBPs) and inhibit its processing to mature cleaved SREBP (SREBP-C), thus blocking its transcriptional activity. One possible mode of the Hippo pathway regulating metabolism is as follows: Hypo-phosphorylated YAP and TAZ accumulate in the nucleus, where they can bind to various TFs (such as HIF1α and SREBP) or TEAD family, to direct transcription and expression of enzymes involved in complex metabolic pathways. AMPK, AMP-activated protein kinase; JCAD, junctional protein associated with coronary artery disease; LATS, large tumor suppressor; MST, mammalian sterile 20 like; OXPHOS, oxidative phosphorylation; SREBP, sterol regulatory element-binding protein; TEAD, TEA domain protein; TFs, transcription factors; YAP/TAZ, yes-associated protein/transcriptional coactivator with PDZ-binding motif.

The development and growth of tumors increase the demands for energy and macromolecules and is often accompanied by the activation of Hippo signaling. Consistent with this, the Hippo signaling pathway and its downstream effectors, YAP and TAZ, have been identified as important regulators of many cellular metabolic pathways of tumor cells, including glucose metabolism, lipid metabolism, amino acid metabolism, and mitochondrial homeostasis [8,60]. The Hippo signaling pathway regulates multiple metabolic pathways (Figure 2), which enables it to coordinate the availability of energy and metabolites to regulate cancer development.

Researchers found that the knockout of MST1/2 and LATS1/2 cells lead to the decrease of glucose levels in the culture medium and the increase of medium acidity, suggesting the increase of glucose uptake and the glycolysis rate, which is consistent with the result of increased YAP/TAZ transcriptional activity [48]. Although there are still limitations at the cellular level, the Hippo signaling pathway has gradually shown its potential as a regulator of intracellular glucose metabolism. As transcriptional coactivators of Hippo signaling, YAP/TAZ were proved to promote glucose uptake and glycolysis by upregulating the expression of glucose transporters and glycolytic enzymes. Studies have identified glucose transporter 3 (GLUT3) and GLUT1 as targets of YAP, with their expression and glucose uptake being regulated by YAP [49,50]. Moreover, our recent study indicated that the activation of YAP promoted the expression of GLUT1 and the glucose uptake of hepatocellular carcinoma (HCC) cells [51]. These studies demonstrate that YAP can promote glucose uptake and glycolysis by upregulating the expression of the glucose transporter. Concurrently, emerging evidences have shown that Hippo signaling regulates glycolysis by regulating the expression of the key enzymes of this pathway [61]. Studies have reported that the deletion of YAP/TAZ in cancer cells could downregulate the expression of a variety of key enzymes involved in glycolysis, including HK1, HK2, PFKFB4, PFKP, GAPDH, PGK1, PGAM1, LDHA, PDHA1, and PDHB, leading to the inhibition of glycolysis activity [52,53]. Consistently, our study also indicated that the knockdown of YAP/TAZ downregulated the transcription and expression of HK2 and LDHA, leading to the decrease of glycolysis activity in HCC cells [51]. In addition to direct transcriptional and expression regulation, YAP/TAZ can also regulate the expression of key glycolysis enzymes by interacting with transcription factors. Hypoxia-inducible factor 1α (HIF1α) and c-Myc as the main transcription factors regulating glycolysis [62,63] were also found to be associated with YAP and to regulate the expression of key glycolysis enzymes and glycolysis. As studies reported, YAP binds to these transcription factors in the nucleus and regulates cell glycolysis [62,64,65,66]. Altogether, Hippo signaling can regulate cell glucose metabolism in a variety of ways.

In addition to glucose metabolism, lipid metabolism and amino acid metabolism are also dysregulated in cancer and can be regulated by Hippo signaling. Sterol regulatory element binding protein (SREBP) is a transcription factor that mainly regulates the biosynthesis of cholesterol, fatty acids, and triacylglycerol, and is involved in the regulation of the expression of key genes in lipid synthesis and absorption [67,68]. It has been demonstrated that YAP is a co-factor of SREBP, and the activation of LATS1 or the inhibition of YAP reduce hepatocyte lipogenesis by inhibiting the function of YAP–SREBP complexes [54]. In addition, as reviewed by Ibar et al., multiple Hippo signaling components (including MST1, LAST2, and YAP) are involved in regulating the activity of SREBPs, thereby controlling lipogenesis and cholesterol synthesis in hepatocyte [8]. Bile acid is an important component of bile and plays an important role in lipid metabolism. A study reported that the activation of Hippo signaling suppressed bile acid metabolism, liver overgrowth, and tumorigenesis [69], suggesting it is involved in the regulation of lipid metabolism. Studies have shown that YAP/TAZ upregulates the expression of amino acid transporter carrier family 38 member 1 (SLC38A1) and solute carrier family 7 member 5 (SLC7A5), resulting in increased amino acid uptake and the regulation of amino acid metabolism in HCC [6]. In addition, the expression of amino acid transporters SLC1A5 and glutaminase1 (GLS1) was also reported to be positively correlated with the expression of YAP/TAZ in human breast cancer samples [55]. Therefore, Hippo signaling is involved in regulating the metabolic network of tumors, but the regulatory mechanism in cancer cells remains to be further explored.

### 4.2. Metabolic Cues That Control Hippo Signaling

It is easy to accept that metabolism is regulated by Hippo signaling, and in fact, studies have made a strong case that Hippo signaling can in turn be regulated by metabolites or metabolic pathways (Figure 2) [70,71]. It is well known that the metabolic reprogramming of cancer cells tends to enhance aerobic glycolysis, and glucose is one of the major sources of cancer cell metabolism. Studies have reported that Hippo signaling is regulated by aerobic glycolysis, and the reduction of glycolysis leads to the inhibition of YAP/TAZ transcriptional activity [8,60,72]. A 2-DG (a glycolysis inhibitor) treatment downregulates the overall levels of the YAP/TAZ gene signature in MCF10A and MDA-MB-231 mammary cells, illustrating that glycolysis regulates YAP/TAZ transcriptional activity [56]. In the absence of glucose, the AMP-activated protein kinase (AMPK) pathway is activated, and AMPK acts as a regulator of the Hippo pathway in response to energy stress, leading to phosphorylation of YAP and promoting its inactivation [49]. These results suggest that glucose and glycolysis are involved in the regulation of the Hippo pathway.

In addition to glucose and glycolysis mentioned above, some other metabolic pathways were also found involved in regulating the activity of Hippo signaling, such as lipids, hormones, and other metabolites [60,73]. As reviewed, alterations on lipid metabolism contribute to the activation of several important oncogenic signaling pathways, including Hippo signaling [73,74]. Research findings have indicated that YAP/TAZ activity is regulated by the SREBP/mevalonate pathway in many cancer cells [54,58]. Oncogenic mutant p53, acting as a positive transcriptional cofactor for SREBPs, leads to increased mevalonic acid and promotes YAP activity in tumor cells [58]. The mevalonate pathway, involved in the synthesis of cholesterol, bile acids, steroid hormones, and statins used to inhibit this pathway, was found to efficiently suppress YAP/TAZ nuclear translocation [58]. Palmitic acid is a common saturated fatty acid in organisms. It has been reported that palmitic acid inhibits YAP by upregulating MST1, thereby inhibiting endothelial cell proliferation, migration, and angiogenesis [75]. These results reveal a tight connection between YAP/TAZ activity and metabolic substances and pathways.

## 5. Target the Hippo Signaling Pathway for Cancer Therapy

As mentioned above, the Hippo pathway is a major signaling pathway that is responsible for human cancer development. The Hippo pathway’s contribution to cancer has sparked interest in the development of potential therapeutics [76]. Given the association of elevated and hyperactive YAP/TAZ with many cancers, the anti-cancer therapeutic strategies targeting the Hippo pathway would aim at inhibiting the activities and functions of YAP and TAZ directly or indirectly [77]. Several Hippo pathway-targeted strategies have been reviewed, such as development of drugs targeting MST and LATS activation, YAP/TAZ activation or YAP/TAZ–TEAD interaction [78,79,80]. The study indicated that YAP activates the DNA damage response pathway, and by targeting YAP, dasatinib acts as a chemosensitizer for a subset of molecular targeted drugs [81]. Apigenin was reported to decrease the expression of YAP/TAZ and disrupt the YAP/TAZ–TEAD interaction in TNBC cells, suggesting a promising therapeutic agent for the treatment of TNBC patients [82]. In addition, statins were identified as potent YAP inhibitors, verteporfin and vestigial like family member 4 (VGLL4) were identified as inhibitors of the YAP–TEAD interaction (as reviewed in [23]). Therefore, some progress has been made in inhibiting upstream Hippo kinase as a strategy for inhibiting tumor progression. However, due to the ambiguous regulatory mechanism of the Hippo pathway, tumor therapies targeting the Hippo pathway still face great challenges.

There are many signal pathways involved in the regulation of cancer development. The regulation of the Hippo pathway by the tumor-related signal pathways is not surprising given its important role in tumorigenesis [83]. The powerful pluripotency of the Hippo pathway in the development of cancer is inseparable from its interaction with a variety of tumor-related signal pathways [84]. Some tumor-related signals, including the Wnt pathway, Notch pathway, Src signal, p53 signal, PI3K/Akt, RAS signaling pathway, TGFβ signaling, among others, were reported to interact with the Hippo pathway and synergistically promote cancer development [83,85,86,87,88,89,90]. They can affect YAP/TAZ dependently/independently of the Hippo pathway, which in turn affects the biological behavior of cancer cells and cancer development. This suggests that the study of the effect of the Hippo pathway on cancer development needs to shift from the concept of the simple linear pathway to the perspective of a network connection composed of multiple signaling pathways. In addition to the direct targeting of Hippo pathway components, pharmacologically regulated signal pathways that interact with the Hippo pathway or combined therapies that inhibit YAP/TAZ target genes may be promising approaches for targeting the Hippo pathway in cancer cells.

## 6. Conclusions

Extensive research studies have provided tremendous insight into the regulation and role of Hippo signaling in cancer development. In response to tissue-level mechanical forces and a variety of biochemical factors, F-actin cytoskeleton acts as the main determinant of the regulation of Hippo–YAP/TAZ activity; through feedback and crosstalk mechanisms, YAP/TAZ influences a variety of cellular events, from metabolism to biological behaviors. At present, some progress has been made in the study of the molecular mechanism by which the cytoskeleton regulates the Hippo signaling pathway, but some key questions remain unanswered [42,91]. The Hippo pathway regulatory network is complex and diverse, and its regulatory mechanism is still poorly understood. A key challenge for the future will be to explore the mechanisms by which the Hippo signaling pathway plays regulatory roles in different environments, and to develop targeted cancer treatments. We believe that targeting the Hippo pathway will lead to fruitful therapies in the near future.

## Figures and Tables

**Table 1:** Integration of Hippo-YAP signaling with metabolism.

Substrates	Targets	Effect	References
**Metabolic Functions Regulated by Hippo signaling Pathway**			
MST1/2 and LATS1/2	unknown	increase glucose uptake and glycolysis	[48]
YAP	GLUT3	Inhibit glucose metabolism	[49]
YAP	GLUT1 and GLUT2	inhibit glucose metabolism	[50]
YAP	GLUT1, HK2 and LDHA	glucose uptake and glycolysis	[51]
YAP/TAZ	GLUT3, HK2, HK1, PFKFB4, PFKP, GAPDH, PGK1, PGAM1, LDHA, PDHA1 and PDHB	glycolysis	[52,53]
LATS1 or YAP	SREBPs	lipogenesis	[54]
MST1, LAST2 and YAP	SREBPs	lipogenesis and cholesterol synthesis	[8]
YAP/TAZ	SLC38A1 and SLC7A5	amino acid metabolism	[6]
YAP/TAZ	GLS1 and SLC1A5	amino acid metabolism	[55]
**Metabolic Cues that Control Hippo signaling**			
PFK1	TEADs	increases YAP/TAZ transcriptional activity	[56]
glucose	YAP	YAP phosphorylation and subcellular localisation	[49]
SCD1	YAP/TAZ	downregulates YAP/TAZ expression, nuclear localization, and activity	[57]
sterols and fatty acids	Mevalonate Pathway	regulate YAP/TAZ activity	[58,59]
amino acids, nucleotides, and lipid molecules	GPCRs	modulate the activity of YAP/TAZ	[47]

## Data Availability

Not applicable.

## Abbreviations

AMPK	AMP-activated protein kinase
ECM	Extracellular matrix
FAK	Focal adhesion kinase
GLUT3	Glucose transporter 3
GPCRs	G-protein-coupled receptors
HCC	Hepatocellular carcinoma
HIF1α	Hypoxia-inducible factor 1α
LATS	Large tumor suppressor
MST	Mammalian sterile 20 like
PFK1	Phosphofructokinase 1
SCD1	Stearoyl-CoA-desaturase 1
SLC38A1	Solute carrier family 38 member 1
SLC7A5	Solute carrier family 7 member 5
Src	Steroid receptor coactivator
SREBP	Sterol regulatory element binding protein
TCGA	The Cancer Genome Atlas
TEAD	TEA domain protein
TFs	Transcription factors
VGLL4	Vestigial like family member 4
YAP	Yes-associated protein
TAZ	Transcriptional coactivator with PDZ-binding motif
